# Drug use by men admitted to a psychiatric hospital*

**DOI:** 10.1590/1518-8345.3370.3296

**Published:** 2020-06-19

**Authors:** Aroldo Gavioli, Patrícia Tieme Nishimura Pazin, Sonia Regina Marangoni, Anai Adario Hungaro, Cleiton José Santana, Magda Lúcia Felix de Oliveira

**Affiliations:** 1 Universidade Estadual de Maringá, Maringá, PR, Brazil.; 2 Instituto de Formação e Prestação de Serviços NSG, Unidade de Terapia Intensiva Adulto, Maringá, PR, Brazil.; 3 Universidade Federal de São Paulo Hospital do Rim e Hipertensão, Fundação Osvaldo Ramos, São Paulo, SP, Brazil.

**Keywords:** Mass Screening, Risk, Substance-Related Disorders, Alcoholism, Street Drugs, Men’s Health, Programas de Rastreamento, Risco, Transtornos Relacionados ao Uso de Substâncias, Alcoolismo, Drogas Ilícitas, Saúde do Homem, Tamizaje Masivo, Riesgo, Trastornos Relacionados con Sustancias, Alcoholismo, Drogas Ilícitas, Salud del Hombre

## Abstract

**Objective:**

to assess risk related to drug use in men admitted to a psychiatric hospital and to identify associations with sociodemographic, socioeconomic variables, and risk conditions.

**Method:**

a cross-sectional study with the application of a screening test in 209 participants hospitalized for mental and behavioral disorders due to the use of psychoactive substances. Statistical analysis was performed using descriptive statistics and adjustment of a binary logistic regression model for moderate or high risk of drug use. The odds ratio measured the strength of association.

**Results:**

high use in life was observed, with alcohol and tobacco experimentation in adolescence. A high prevalence of related risk was observed for alcohol, tobacco, smoked and inhaled cocaine, and marijuana. Moderate and elevated risks were found for tobacco (22.5% and 62.5%, respectively), alcohol (13.5% and 73%), marijuana (16% and 32.5%), smoked cocaine (3% and 41%) and inhaled cocaine (9% and 19.5%).

**Conclusion:**

the results showed high use in life, with an age of early experimentation. Tobacco and alcohol are the main drugs used by hospitalized men.

## Abstract (pt)

**Objetivo:**

avaliar risco relacionado ao uso de drogas em homens internados em hospital psiquiátrico e identificar associações com variáveis sociodemográficas, socioeconômicas e condições de risco.

**Método:**

estudo transversal com aplicação de teste de rastreamento em 209 participantes internados por transtornos mentais e comportamentais, devido ao uso de substâncias psicoativas. A análise estatística foi realizada através de estatísticas descritivas e ajuste de modelo de regressão logística binária para o risco moderado ou elevado relacionado ao uso de drogas. A força de associação foi medida pela razão de chances.

**Resultados:**

observou-se elevado uso na vida, com início de experimentação de álcool e tabaco ainda na adolescência. Maiores prevalências de risco relacionado observadas para álcool, tabaco, cocaína fumada e inalada, e maconha. Riscos moderados e elevados foram encontrados para tabaco (22,5 e 62,5%, respectivamente), álcool (13,5 e 73%), maconha (16 e 32,5%), cocaína fumada (3 e 41%) e inalada (9 e 19,5%).

**Conclusão:**

os resultados apontaram elevado uso na vida, com idade de experimentação precoce. O tabaco e o álcool são as principais drogas utilizadas pelos homens internados.

## Abstract (es)

**Objetivo:**

evaluar riesgo relacionado al uso de drogas en hombres internados en hospital siquiátrico e identificar asociaciones con variables sociodemográficas, socioeconómicas y condiciones de riesgo.

**Método:**

estudio transversal con aplicación de test de rastreo en 209 participantes internados por trastornos mentales y conductuales debido al uso de sustancias psicoactivas. El análisis estadístico fue realizado a través de la estadística descriptiva y ajuste del modelo de regresión logística binaria, para el riesgo moderado o elevado de tipo uso drogas. La fuerza de asociación fue medida por la razón de chances.

**Resultados:**

se observó elevado uso en la vida, con inicio de experimentación de alcohol y tabaco en la adolescencia. Fueron observadas mayores prevalencias de riesgo relacionado para alcohol, tabaco, cocaína fumada e inhalada, y mariguana. Riesgos moderados y elevados fueron encontrados para tabaco (22,5% y 62,5%, respectivamente), alcohol (13,5% y 73%), mariguana (16% y 32,5%), cocaína fumada (3% y 41%) e inhalada (9% y 19,5%).

**Conclusión:**

los resultados apuntaron elevado uso en la vida, con edad de experimentación precoz. El tabaco y el alcohol son las principales drogas utilizadas por los hombres internados.

## Introduction

The Anti-Asylum Fight in Brazil resulted in the enactment of Law 10,216, of April 6^th^, 2001^(^ 1 ^)^, creating barriers to involuntary and compulsory hospitalizations, especially in nursing homes. In 2011, Psychosocial Care Networks (RAPS, its acronym in Portuguese) was created aimed at people suffering from crack, alcohol and other drugs, changing the approach of the user and the drug addict, who, before being criminalized, becomes a health disorder patient, and differentiated strategies for care and social reintegration must be adopted for these users or drug addicts^(^ 2 ^)^.

Hospital care for drug users and addicts has been gradually replaced by non-hospital care based on rehabilitation and social reintegration, through RAPS articulated to other health networks^(^ 3 ^)^. These advances were positive, but the comprehensive treatment of drug addicts is incipient and hampered by the low role of men in relation to health care, the social stigma of drug use and dependence, and the current assistance gap in Primary Care^(^ 4 ^)^. Such factors can lead to late demand for treatment, increasing the indicators of morbidity and mortality and costs to the health system^(^ 3 ^)^.

Disorders related to drug use are considered a public health problem, with severe personal and community consequences and high worldwide prevalence, contributing substantially to the overload^(^ 5 ^)^ of public health and high economic costs by combating crime, losses, productivity, and health care costs. Despite public policies aimed at this problem, patients have high variability in response to interventions and high rates of relapse^(^ 6 ^-^ 7 ^)^.

Knowing the sociodemographic and clinical profiles of hospitalized drug users and addicts contributes to the deepening of the knowledge of this theme for the area of public health and nursing. Despite its importance, few studies by Brazilian nurses focus on the diagnostic criteria for hospitalization of men in a psychiatric hospital for diagnosing disorders related to substance use. Studies in this area could allow the generation of subsidies for coping plans consistent with the needs of these clients, as well as promoting public policies aimed at prevention, early diagnosis and treatment.

This study aimed to assess risk related to drug use in men admitted to a psychiatric hospital and to identify associations with sociodemographic, socioeconomic variables and risk conditions.

## Method

A cross-sectional and descriptive study carried out in a psychiatric hospital integrated with RAPS, with 364 beds, in a municipality in the northwest of the State of Paraná. Data were collected in the inpatient unit for male patients with drug dependency, which has 80 beds. The study population consisted of patients admitted to this unit.

Sampling was defined in a non-probabilistic way, and individuals were selected by convenience. In the months before data collection, the occupancy rate above 90% was observed, and, in 2015, 717 hospitalizations were checked in the unit that holds drug addicts. For the sample calculation, the number 717 was considered as the size of the population, estimating that 80% of the patients would present risk related to the use (RRU) of drugs at moderate or high levels for at least one type of drug. By considering a margin of error of 5% and a confidence level of 95%, 184 interviews would be necessary to represent this universe. Thus, it was decided to recruit for the study the 218 patients hospitalized in the period between April and July 2016.

The inclusion criterion was the admission of a male patient who was diagnosed with disorders related to substance use with coding F10 to F19 according to the 10^th^ version of the International Statistical Classification of Diseases and Related Health Problems (ICD 10). Those hospitalized patients with abstinence syndrome, delirium or any other clinical condition that impaired them from answering to the interview or signing the Informed Consent Form (ICF) were excluded.

During the data collection, the researchers checked the new admissions to the unit for drug addicts three times a week, and carried out the approach of patients and their availability to participate in the research. After a brief explanation of the research objectives, the patients were interviewed in a private place.

The structured interview script consisted of a block of sociodemographic, socioeconomic and risk conditions variables, and the RRU’s tracking instrument for drugs Alcohol, Smoking and Substance Involviment Screening Test, version 3.1 (ASSIST 3.1).

For sociodemographic characterization, the following variables were used: age, marital status, number of children, race/color, and religion. As for the socioeconomic characterization, the following were assessed: family income, if the participant had a job at the time of the research, housing conditions, schooling, and data related to the risk conditions to which he is exposed due to drug use, being, for this, the variables listed were driving under the influence of alcohol or other drugs, involuntary hospitalization, unprotected sexual behavior or with multiple partners, living with family members who use drugs and court problems.

ASSIST 3.1 consists of eight questions about the use of nine classes of substances (tobacco, alcohol, marijuana, cocaine, amphetamines, sedatives, inhalants, hallucinogens, and opiates) and aims to address the frequency of use of each of them (throughout life and in the last three months) using the variables: problems related to use; concern on the part of people close to the user; impairment in performing expected tasks; unsuccessful attempts to cease or reduce use; feeling of compulsion; and injectable use. Each answer corresponds to a score, which ranges from zero to 8, and the sum is the risk score related to drug use (RRU), which can vary from zero to 39^(^ 8 ^-^ 9 ^)^.

For alcohol use, RRU scores in the range of zero to 10 are considered low risk; from 11 to 26, as of moderate risk; and, when higher than 27 points, high risk. For other drugs, the scores needed to fill each category are, respectively, zero to 3 points, 4 to 26 points, and higher than 27 points. Many problems associated with drug use can be identified, including acute intoxication, chronic use or addiction, and high risk of injecting substance use. A result with RRU scores in the middle range of ASSIST is indicative of the use of dangerous or harmful substances (moderate risk), and higher scores point drug dependence (high-risk)^(^ 8 ^-^ 10 ^)^.

The constant use of injectable drugs in the last three months is classified by the ASSIST 3.1 instrument as high-risk, with no score calculation for this question. Depending on the use pattern, users with constant use should undergo brief intervention, more in-depth intervention and/or intensive treatment.

The data were compiled using the Statistical Package for Social Sciences (SPSS) software, and the independent variables (sociodemographic, socioeconomic and risk conditions) were dichotomized for further performance of the descriptive statistical analysis and adjustment of the logistic regression of binary model for the moderate or high RRU event, separately for each drug. The selection of independent variables for the final model was performed using the backward stepwise method with a significance level of 5%. For the significant variables in the final model, the odds ratio (OR) and its respective 95% confidence interval (95% CI) were adopted as an association measure^(^ 11 ^)^.

The study met national and international ethical standards in research involving human beings, and the study was submitted to the approval of the Human Research Ethics Committee of Faculdade Ingá/Uningá, under number 51754915.5.0000.5220, being approved by opinion number 1.375. 463.

## Results

In the study period, there were 218 hospitalizations in the drug addicts’ sector, and 209 men interviews were carried out, with the six losses of individuals who were experiencing abstinence syndrome or inability to sign the IC, and three refusals to participate in the research. The data on the sociodemographic, socioeconomic, and risk conditions of the sample are shown in Table 1.

**Table 1:** Sociodemographic, socioeconomic variables and risk conditions of the 200 men admitted to a psychiatric hospital. Maringá, PR, Brazil, 2016

Categories	n (%)
Sociodemographic	
Age, years	
18-34	107 (51.5)
35-62	102 (48.5)
Marital status	
Single	165 (79.0)
Married/ in a domestic partnership	44 (21.0)
Children	
No children	82 (39.3)
With children	127 (60.7)
Race/color	
White	101 (48.4)
Black	30 (14.3)
Brown	78 (37.3)
Religion	
Catholic	91 (43.5)
Evangelicals	74 (35.4)
Other religions	10 (4.8)
None	34 (16.3)
Socioeconomic	
Family income	
Up to R$1,600.00	102 (48.8)
> R$1,600.00	107 (51.2)
Currently working	
Yes	101 (48.4)
No	108 (51.6)
Housing	
Home owner	108 (51.6)
Not owner	101 (48.4)
Schooling	
None	2 (1.0)
Incomplete primary	39 (18.7)
Complete primary	89 (42.6)
Complete elementary school	39 (18.7)
High School	36 (17.2)
Higher education	4 (1.9)
Risk conditions	
Driving under the influence of alcohol/ drugs	
Yes	140 (70.0)
No	69 (33.0)
Compulsory hospitalizations	
Yes	162 (77.5)
No	47 (22.5)
Unprotected sexual behavior	
No	26 (12.4)
Yes	183 (87.6)
Family members who use drugs	
No	26 (12.4)
Yes	183 (87.6)
Court problems	
No	86 (41.1)
Yes	123 (58.9)

The interviewees (51.5%) were between 18 and 34 years old, with an average of 34.9 years (standard deviation - SD = 10.87 years) and median of 34 years; single (79%), with children (60.7%) and white/race (48.4%). The socioeconomic data showed an average monthly family income of R$ 1,997.93 (SD = 1,903.68) and a median of R$ 1,600.00. A total of 51.5% of men were not working at the time of data collection, lived in their own home (51.5%) and had less than 8 years of study (62.3%) (Table 1).

In the sample, it was observed that men were hospitalized for disorders related to substance use on average 4.33 times (SD = 4.9), with a median of hospitalizations of 2 and mode of hospitalization, with 27.2% (n: 57) were hospitalized for the first time.

Regarding the risk conditions they were subjected to in drug use, most reported having driven under the influence of some drugs (70%), being hospitalized voluntarily (77.5%), presenting sexual risk behavior (87,6%), lived with at least one family member who was a user of some drugs (87.6%) and had court problems (58.9%) (Table 1).

The use of drugs in life, screened by the ASSIST instrument, is shown in Table 2. Three patterns of prevalence of lifetime use and age of drug experimentation were evidenced: the first was related to legal drugs alcohol and tobacco, which presented higher prevalence of use in life and young ages of experimentation. The second pattern concerns illicit drugs (marijuana, inhalants, cocaine and crack), for which the average ages of experimentation was in late adolescence and early adulthood, and which have intermediate prevalence of use in life. Finally, there were recreational drugs, such as hallucinogens, amphetamines and opioids, with lower prevalence of lifetime use and higher mean ages of experimentation, prevalent in young adults. In the sample, no hypnotic and sedative drugs were used in life. It was also observed that 3 men (1.4%) reported having already used injectable cocaine, but experimentally, without, however, constant use (Table 2).

**Table 2:** Lifetime use of drugs of abuse tracked by ASSIST 3.1* and mean age of drug abuse testing of 209 men admitted to a psychiatric hospital. Maringá, PR, Brazil, 2016

Drug type	Average age of experimentation in years (Standard Deviation)	Lifetime use n (%)
Tobacco	13.3 (3.4)	190 (91.0)
Alcohol	14.8 (3.8)	197 (94.3)
Marijuana	16.4 (6.4)	153 (73.2)
Inhalants	17.1 (5.5)	87 (41.6)
Cocaine	18.1 (4.9)	115 (55.0)
Crack	19.9 (6.3)	114 (54.5)
Hallucinogens	20.2 (6.0)	45 (21.5)
Amphetamines	19.6 (5.2)	26 (12.4)
Opioids	23.5 (6.5)	12 (5.7)
Injectable use (experimental)	-	3 (1.4)

* ASSIST 3.1 = Alcohol, Smoking and Substance Involvement Screening Test version 3.1

The data related to the drug RRU are shown in Table 3. For tobacco, 85.2% of the participants were classified as moderate and high-risk. For alcohol, 86.6%. As for illicit drugs, the prevalence of RRU was: marijuana for 48.3% of the sample; inhaled cocaine for 32.5%, for smoked cocaine (crack), 44.0% of men were into moderate and high-risk levels. Regarding amphetamines, the prevalence of moderate and high RRU was 3.3%; for inhalants, it was 3.0%; for hallucinogens, it was 8.6%; and, finally, the prevalence of the RRU of opioids was 1.4%. In the present sample, a 6.2% prevalence of injectable drug use (cocaine) was observed in the last 3 months, regularly, in which this pattern of use was considered high-risk according to ASSIST 3.1.

**Table 3:** Risk related to the use (RRU) of drugs screened by the Alcohol, Smoking and Substance Involvement Screening Test, version 3.1 (ASSIST 3.1) in 209 men admitted to a psychiatric hospital. Maringá, PR, Brazil, 2016

Drug type	RRU* score according to ASSIST 3.1^†^			
	Low n (%)	Moderate n (%)	High n (%)	Score mean ASSIST3.1^†^ (SD^‡^)
Tobacco	31 (14.8)	47 (22.5)	131 (62.7)	20.0 (9.7)
Alcohol	28 (13.4)	28 (13.4)	153 (73.2)	27.5 (12.8)
Marijuana	108 (51.7)	33 (15.8)	68 (32.5)	11.8 (14.6)
Inhalants	201 (96.2)	4 (1.9)	4 (1.9)	0.7 (3.6)
Cocaine	150 (71.8)	19 (9.1)	40 (19.1)	7.4 (13.4)
Crack	117 (56.0)	6 (2.9)	86 (41.1)	15.0 (17.4)
Hallucinogens	191 (91.4)	11 (5.3)	7 (3.3)	1.0 (4.2)
Amphetamine	202 (96.7)	4 (1.9)	3 (1.4)	0.4 (3.2)
Opioids	206 (98.6)	3 (1.4)	0 .0	0.12 (1.0)
Injectable drugs^§^	196 (93.8)	-	13 (6.2)^||^	na^¶^

* RRU = Risk related to use;

† ASSIST 3.1 = Alcohol, Smoking and Substance Involvement Screening Test version 3.1;

‡ SD = Standard deviation;

§ Injectable drugs = Injectable cocaine;

|| Use in the last 3 months represents a high-risk level;

¶ not applicable

Regarding the association of drugs, that is, the concomitant use of psychoactive substances that had a prevalence of RRU, at moderate and high levels, it was observed that 72.7% of hospitalized men were positively screened for tobacco and alcohol, 29.7% for tobacco, alcohol and marijuana, 18.1% for tobacco, alcohol, marijuana and cocaine, 11.4% for tobacco, alcohol, marijuana, cocaine and crack, 1.9% for tobacco, alcohol, marijuana, cocaine, crack and amphetamines and 1.0% for all 7 substances (Table 4).

**Table 4:** Distribution of drug association frequencies screened by the Alcohol, Smoking and Substance Involvement Screening Test, version 3.1 (ASSIST 3.1) in 209 men admitted to a psychiatric hospital. Maringá, PR, Brazil, 2016

Variables	Drug type	n (%)
Association type	Tobacco and alcohol	152 (72.7)
	Tobacco, alcohol and marijuana	62 (29.7)
	Tobacco, alcohol, marijuana and cocaine	38 (18.1)
	Tobacco, alcohol, marijuana, cocaine and crack	24 (11.4)
	Tobacco, alcohol, marijuana, cocaine, crack and amphetamines	4 (1.9)
	All tracked substances	2 (1.0)

The result of the binary logistic regression of the variables (sociodemographic, socioeconomic and risk conditions) on the RRU outcome (moderate and high) is shown in Table 5. There were no significant associations between the variables and the RRU levels of tobacco, alcohol, amphetamines, inhalants, hypnotics/sedatives and opioids and injecting drugs.

**Table 5:** Binary logistic regression analysis for the effect of the selected variables on the score of risk related to moderate or high use of each type of drugs in men admitted to a psychiatric hospital. Maringá, PR, Brazil, 2016

Variables	n (%)	OR* (IC 95%)^†^
Marijuana		
Age 18 to 34 years	69 (33.0)	5.752 (2.873-11.518)
Drug use by family members: yes	75 (35.8)	2.430 (1.164-5.071)
Unprotected sexual behavior: yes	53 (25.3)	2.119 (1.091-4.115)
Court problems	74 (35.4)	2.924 (1.444-5.921)
Cocaine		
Age 18 to 34 years	46 (22.0)	7.065 (3.071-16.254)
Drug use by family members: yes	44 (21.0)	3.414 (1.520-7.666)
No religion	17 (8.1)	3.234 (1.334-7.839)
Unprotected sexual behavior: yes	36 (17.2)	2.746 (1.294-5.828)
Crack		
Age 18 to 34 years	64 (30.6)	3.225 (1.580-6.583)
Court problems	70 (33.5)	3.257 (1.573-6.744)
Voluntary hospitalization: no	13 (6.2)	2.657 (1.131-6.241)
Number of hospitalizations > 2	53 (25.3)	2.447 (1.239-4.833)
Hallucinogens		
Age 18 to 34 years	10 (4.7)	5.004 (1.037-24.153)
Currently working: no	2 (1.0)	5.638 (1.170-27.164)

* OR = Odds ratio;

† CI = 95% confidence interval

Regarding the moderate and high-risk scores for marijuana, there was a significant association with having court problems, risky sexual behavior, age between 18 and 34 years old, and living with drug users.

For inhaled cocaine RRU scores, at moderate and high levels, there was a statistically significant association with the variables not having a religion, having risky sexual behavior, being aged 18 to 34 years, and living with drug users. For smoked cocaine (crack), RRU scores were statistically associated with the variables having court problems, being involuntarily hospitalized, age between 18 and 34 years old, and having been hospitalized more than twice.

For hallucinogenic drugs, there were significant associations, at the 95% confidence level between the moderate and high scores of RRU with the variables not currently working and age 18 to 34 years.

## Discussion

There were four main findings in this study: the sociodemographic profile; the experimentation age (use in life); the RRU scores of drugs; and the association of sociodemographic variables with the RRU levels of marijuana, cocaine, crack and hallucinogens.

Known to be greater among men, the beginning of drug use is also earlier, associated to constant use. Factors such as easy access, low cost and preference for drugs considered potent, are pointed out as contributing to this circumstance. This scenario results in difficulties for recovery, being a challenge for health professionals who will need to face the low adherence to the proposed treatments and the high rates of relapses^(^ 12 ^-^ 14 ^)^. Data on this fact are important to advocate health and de-institutionalization policies for these men.

Drug experimentation during adolescence, that is, from 12 to 18 years old^(^ 15 ^)^, is virtually normative. However, most individuals do not become dependent on drugs^(^ 12 ^)^. The vulnerabilities for the development of drug-related disorders are related to the age of experimentation, genetics, gender and psychiatric causes, such as depression, conduct disorders, Attention Deficit Hyperactivity Disorder (ADHD) - classified as intrinsic; extrinsic are access to drugs, the environment (family, school and community), companions, living with user parents, stress common to this development stage and physical or sexual abuse^(^ 12 ^-^ 14 ^)^.

Studies point that drug-related disorders are highly prevalent worldwide, with tobacco and alcohol abuse being the most prevalent^(^ 5 ^)^, causing high costs to public systems (health, safety and social security) related to treatment and the direct consequences of abuse, since drugs play an important role with certain types of cancer, respiratory, cardiovascular, gastrointestinal diseases and external causes of morbidity and mortality, such as accidents, violence and crime^(^ 16 ^)^.

The global burden of diseases caused by drug use is distinct influenced by sociodemographic and socioeconomic factors. It is more prevalent in countries with low and medium incomes, as is the case in Brazil, and, depending on this characteristic, we observed that the hospitalized men were young or young adults (of economically active age), single, with children, with less than 8 years of study and who did not work at the time of the research, pointing that drug use is a global social problem that varies substantially between countries, depending on the social development of each country^(^ 5 ^,^ 17 ^)^.

Drugs are responsible for 14.7% of the disability-adjusted life years lost, and this significance will continue to grow as the transition in the burden of communicable to non-communicable diseases occurs. Health systems around the world need to respond to this growing demand by implementing proven and economically feasible interventions, as well as supporting the research needed to develop better prevention and treatment options^(^ 18 ^)^.

The RRU risk of alcohol and tobacco were the most prevalent in the sample, and tobacco, which does not lead to psychiatric hospitalizations. These substances are risk factors for several non-communicable diseases, such as lung cancer and other types of cancer, chronic obstructive pulmonary disease (COPD), cardiovascular, gastrointestinal, and psychiatric diseases. A population-based study in a city in southern Brazil showed that among the main risk factors for non-communicable diseases are smoking, present in 16.1% of the population, and alcoholism, present in 23.4%, classified as heavy drinking^(^ 18 ^)^.

Alcohol intoxication and drug use lead the user to increase the probability of doing or getting involved in something potentially harmful^(^ 19 ^)^, such as drunk driving, violent behaviors, and crimes^(^ 20 ^)^, or negligent behavior (sexual, for example)^(^ 21 ^)^. About activities, they can increase the probability of accidents^(^ 22 ^)^, leading to an increased risk of injuries and traumas^(^ 17 ^)^.

The multiple drug use found in the sample corroborates the findings of other studies^(^ 23 ^-^ 24 ^)^, in which a trend of multiple drug use as observed in hospitalized individuals for the treatment of alcoholism^(^ 23 ^)^. In those hospitalized for crack addiction, involvement with legal drugs was observed during adolescence^(^ 24 ^)^.

The assistance of drug users is affected by the media, resulting in speeches that simultaneously refuse police repression and the use of evident coercion apparatus, but are consistent with institutional devices that are not always evident of sanitary violence, such as the hospitalization of these users, accepted by liabilities recognition of the disarticulation of RAPS and the biomedical staff shared by professionals^(^ 25 ^)^.

The user is defined as non-volitive, dominated by the autonomous drug. Such practices corroborate the lack of assistance in Primary Care, legitimizing care gaps, filled by more humanized, but no less perverse, institutions that survive sustained through public funding, adjusting to the weaknesses in the implementation of psychiatric and health reform in Brazil^(^ 25 ^)^.

The fact that the data collection took place within one psychiatric institution can be considered as limitations of this study, which may distort the responses of the participants, unsettled about hospital discharge or even fearful of the theme regarding illegality and, ultimately, due to the stigma of the participants themselves. However, this approach has strengths and brings as a contribution to the advancement of knowledge the possibility of highlighting the sociodemographic, socioeconomic profiles and risk conditions that influence the drug RRU, which can be used to support planning and policy-making prevention programs, harm reduction programs, as well as contributing to training primary care to assist these individuals.

## Conclusion

There was a risk related to use at moderate and high scores for alcohol, tobacco, marijuana, crack, hallucinogens, amphetamines, inhalants, and opioids. In the sample, it was observed that 1.4% of men tried (lifetime use) injectable cocaine without keeping constant use, and 6.2% make regular use (current use) in the evaluation of the last 3 months.

The variable age from 18 to 34 years was associated with the risk related to the use of marijuana, inhaled and smoked cocaine and hallucinogens; living with drug users was associated with the risk related to the use of marijuana and inhaled cocaine; getting involved in court problems was associated with the risk related to the use of alcohol, marijuana, and smoked cocaine; the variable having risky sexual behavior was associated with the risk related to the use of marijuana and inhaled cocaine; not having a religion was associated with the risk related to use inhaled cocaine; involuntary hospitalization and having been hospitalized more than twice were associated with the risk related to the use of smoked cocaine, and not currently working was associated with the risk related to the use of hallucinogens.
